# The classification of the bladder cancer based on Vision Transformers (ViT)

**DOI:** 10.1038/s41598-023-47992-y

**Published:** 2023-11-24

**Authors:** Ola S. Khedr, Mohamed E. Wahed, Al-Sayed R. Al-Attar, E. A. Abdel-Rehim

**Affiliations:** 1 Department of Mathematics -Computer Science, Faculty of Science, Suez Canal University, Ismailia, 44745 Egypt; 2 Department of Computer Science, Faculty of Computers and Informatics, Suez Canal University, Ismailia, 44692 Egypt; 3 Department of Pathology, Faculty of Vetrinary Medicine, Zagazig University, Zagazig, 11144 Egypt; 4 Department of Mathematics, Faculty of Science, Suez Canal University, Ismailia, 41552 Egypt

**Keywords:** Medical research, Mathematics and computing

## Abstract

Bladder cancer is a prevalent malignancy with diverse subtypes, including invasive and non-invasive tissue. Accurate classification of these subtypes is crucial for personalized treatment and prognosis. In this paper, we present a comprehensive study on the classification of bladder cancer into into three classes, two of them are the malignant set as non invasive type and invasive type and one set is the normal bladder mucosa to be used as stander measurement for computer deep learning. We utilized a dataset containing histopathological images of bladder tissue samples, split into a training set (70%), a validation set (15%), and a test set (15%). Four different deep-learning architectures were evaluated for their performance in classifying bladder cancer, EfficientNetB2, InceptionResNetV2, InceptionV3, and ResNet50V2. Additionally, we explored the potential of Vision Transformers with two different configurations, ViT_B32 and ViT_B16, for this classification task. Our experimental results revealed significant variations in the models’ accuracies for classifying bladder cancer. The highest accuracy was achieved using the InceptionResNetV2 model, with an impressive accuracy of 98.73%. Vision Transformers also showed promising results, with ViT_B32 achieving an accuracy of 99.49%, and ViT_B16 achieving an accuracy of 99.23%. EfficientNetB2 and ResNet50V2 also exhibited competitive performances, achieving accuracies of 95.43% and 93%, respectively. In conclusion, our study demonstrates that deep learning models, particularly Vision Transformers (ViT_B32 and ViT_B16), can effectively classify bladder cancer into its three classes with high accuracy. These findings have potential implications for aiding clinical decision-making and improving patient outcomes in the field of oncology.

## Introduction

Bladder cancer is a major global health concern, contributing significantly to cancer-related morbidity and mortality. Accurate classification of its distinct histopathological subtypes is crucial for tailored treatments and predicting disease progression^1^. Bladder cancer is a prevalent global malignancy, presenting a significant public health challenge. According to the World Health Organization (WHO), it ranks among the top ten most frequently diagnosed cancers, with approximately 550,000 new cases reported annually worldwide^2^. The disease exhibits diverse histopathological subtypes, including invasive, non-invasive, and normal tissue, necessitating tailored therapeutic approaches. Precise and dependable classification of these subtypes is critical for determining appropriate treatment modalities, such as surgery, radiation therapy, or immunotherapy, while also assessing the likelihood of disease progression and recurrence^3^.

Bladder cancer commonly originates from the bladder's inner surface epithelium (urothelium), with urothelial carcinomas being the predominant type. However, a subset of bladder cancers (10–25% of cases) displays variant histology, exhibiting distinct histomorphological phenotypes such as squamous cell carcinoma, small-cell carcinoma, and adenocarcinoma. High-grade urothelial carcinomas can manifest as micropapillary, sarcomatoid, plasmacytoid, nested, or microcystic variants, often showing divergent differentiation into squamous and glandular histologies. Bladder cancers with variant histology tend to be locally aggressive, prone to metastasis, and exhibit poor responses to existing therapies. Nonetheless, there is ongoing debate regarding the true impact of histology on patient outcomes^4^.

Moreover, accurate bladder classification contributes to optimizing patient care by facilitating early detection of aggressive tumors and enabling personalized medicine, ultimately leading to enhanced survival rates and improved quality of life for individuals afflicted by this multifaceted ailment. As the incidence of bladder cancer continues to rise, advancements in precise classification methodologies, including the incorporation of sophisticated deep learning techniques, hold the potential to revolutionize diagnostic and therapeutic approaches, ultimately culminating in better patient outcomes and alleviating the global burden of bladder cancer^5^.

Deep learning, a specialized branch of machine learning, employs intricate neural networks to decipher intricate patterns in extensive datasets. In the realm of medical imaging, these deep learning algorithms have played a pivotal role in refining the precision and effectiveness of various tasks, such as segmenting images, recognizing objects, and extracting essential features^6^. This paper likely explores the application of deep learning models to bladder datasets, potentially employing techniques like Transfer learning model to automatically discern and extract pertinent contextual information from medical images. This process might involve the identification of specific structures within the bladder, the detection of irregularities or deviations from the norm, and the provision of valuable insights crucial for accurate diagnosis and meticulous treatment planning.

This paper presents a comprehensive study on the classification of bladder cancer into its three distinct classes, leveraging cutting-edge deep learning methodologies. Histopathological analysis remains the gold standard for diagnosing and subclassifying bladder cancer due to its ability to assess cellular morphology and tissue architecture, crucial factors in determining tumor behavior. Nevertheless, the subjectivity inherent in manual interpretation has motivated the exploration of data-driven approaches aimed at enhancing diagnostic accuracy and reproducibility.

Our primary objective is to evaluate the performance of state-of-the-art deep learning models in classifying bladder cancer subtypes. We focus on two categories of models: transfer learning and Vision Transformers (ViT), both of which have demonstrated remarkable success in various image recognition tasks. Transfer learning capitalizes on pre-trained knowledge from large-scale datasets to overcome data limitations and expedite model convergence, while Vision Transformers, a relatively novel paradigm, enable efficient image processing through self-attention mechanisms, making them particularly suitable for medical image analysis. Through rigorous evaluation, six deep learning models were examined, including EfficientNetB2, InceptionResNetV2, InceptionV3, ResNet50V2, and two Vision Transformer variants, ViT_B32 and ViT_B16. We aim to identify the most effective model for multi-class bladder cancer classification. To ensure robust model performance and generalizability, the dataset used in this study comprises a diverse range of bladder tissue samples, meticulously annotated into training, validation, and test sets.

Our work contributes to the growing body of literature on artificial intelligence in healthcare, emphasizing the pivotal role of advanced technologies in shaping the future of precision medicine. The following points conclude the contributions of this research:

1. *Comprehensive evaluation* We assess six advanced deep learning models, including transfer learning architectures and Vision Transformers, for multi-class bladder cancer classification, providing valuable insights for informed diagnostic decisions. The dataset is manually collected from the Faculty of Medicine, Zagazig University.
2. *Advancing bladder cancer classification* Leveraging advanced deep learning, we improve bladder cancer classification, enabling precise identification of invasive, non-invasive, and normal tissue subtypes for better treatment planning and prognostic capabilities.
3. *Vision transformers' potential* Exploring Vision Transformers' success in bladder cancer classification highlights their high accuracy and suitability for medical image analysis, encouraging further investigation in medical imaging tasks.
4. *Addressing subjectivity* Employing deep learning reduces inter-observer variability in conventional diagnostics, yielding more objective and reproducible classification, and enhancing bladder cancer diagnosis reliability.

## Related works

In recent years, the automatic feature extraction capabilities of deep learning-based models have made them more and more well-liked in the field of medical image analysis. Convolutional neural networks (CNNs) stand out among these models as the most popular and well-regarded framework, notably for classification problems requiring radiological data. For instance, researchers in Yang et al.^7^, categorize muscle-invasive bladder cancer (MIBC) and non-muscle-invasive bladder cancer (NMIBC) using contrast-enhanced CT (CECT) images, a comprehensive collection of nine CNN-based models was created. 369 patients who underwent radical cystectomy provided the 1200 CT scans that made up the dataset utilized for training and evaluation. 249 of these individuals had NMIBC diagnoses, and the remaining 120 had MIBC. The CNN model was initially trained using the ImageNet dataset to improve classification performance. The result of the model are AUC equal to 99.70% and accuracy of 93.90%. Additionally, Chapman-Sung et al.^8^ developed a CNN-based classification scheme for the two stages of bladder cancer. 84 bladder cancer CR urography (CTU) images from 76 patients were included in the training dataset, with 41 CTUs containing high-stage cancer and 43 CTUs including low-stage cancer. 90 bladder CTUs from 86 patients made up the test set. On the same dataset, the texture-based classification method utilizing SVM attained an accuracy of 88.00%, while the CNN classifier displayed a high-test set prediction accuracy of 91.00%. According to Chapman-Sung et al.^9^, The authors contend that automatically generated features using CNN-based classifiers are outperformed by feature extraction based on domain expertise. The goal was to categorize two difficult early bladder cancer stages, Ta (non-invasive) and T1 (superficially invasive), which are tricky to distinguish histologically. 1177 bladder scans totaled in the dataset, of which 460 were classified as minimally invasive and 717 as superficially invasive. The accuracy of the CNN classifiers, which was just 84.0%, was much lower than that of supervised machine learning classifiers developed using manually extracted features. Also other researchers in Sarkar et al.^10^ planned to use the radionics-assisted interpretation of CT scans to develop a diagnosis model for bladder cancer. Normal vs bladder cancer, NMIBC versus MIBC, and post-treatment changes (PTC) versus MIBC were the three classification tasks that were carried out. The dataset had 165 areas of interest (ROIs), 100 normal samples, and 65 cancer samples. It was retrospective and single-center. Cross-validation was performed ten times. The accuracy for the LDA classifier on XceptionNet-based characteristics for determining whether a person has cancer or not was 86.07%. Researchers in the study^11^ collected CT data from 75 bladder patients for classification and staging using a ResNet-based model, and applied super-resolution to enhance medical pictures. The model has 94.74% sensitivity rate, which is comparable to preoperative pathological diagnosis, was attained after retrospectively evaluating data from 76 individuals with bladder cancer. The 183 patients that made up the dataset utilized in the study^12^ were divided into three sets: 110 for training, 73 for internal validation, and 75 for external testing. The researchers created a brand-new convolutional network called FGP-Net that included DFL and Dense Blocks modules. The FGP-Net obtained an AUC (Area Under the Receiver Operating Characteristic Curve) of 0.861 and an accuracy of 0.795 when tested on the internal dataset. The FGP-Net achieved an AUC of 0.791 and an accuracy of 0.747 on the external dataset. These results demonstrate the effectiveness of the FGP-Net in classifying the patients and its generalizability on an external dataset.

These studies collectively demonstrate the promising potential of deep learning-based models in bladder cancer classification, offering valuable insights into the development of sophisticated approaches for improving diagnostic accuracy and patient outcomes in the field of oncology.

## Material and methods

**Figure 1:**
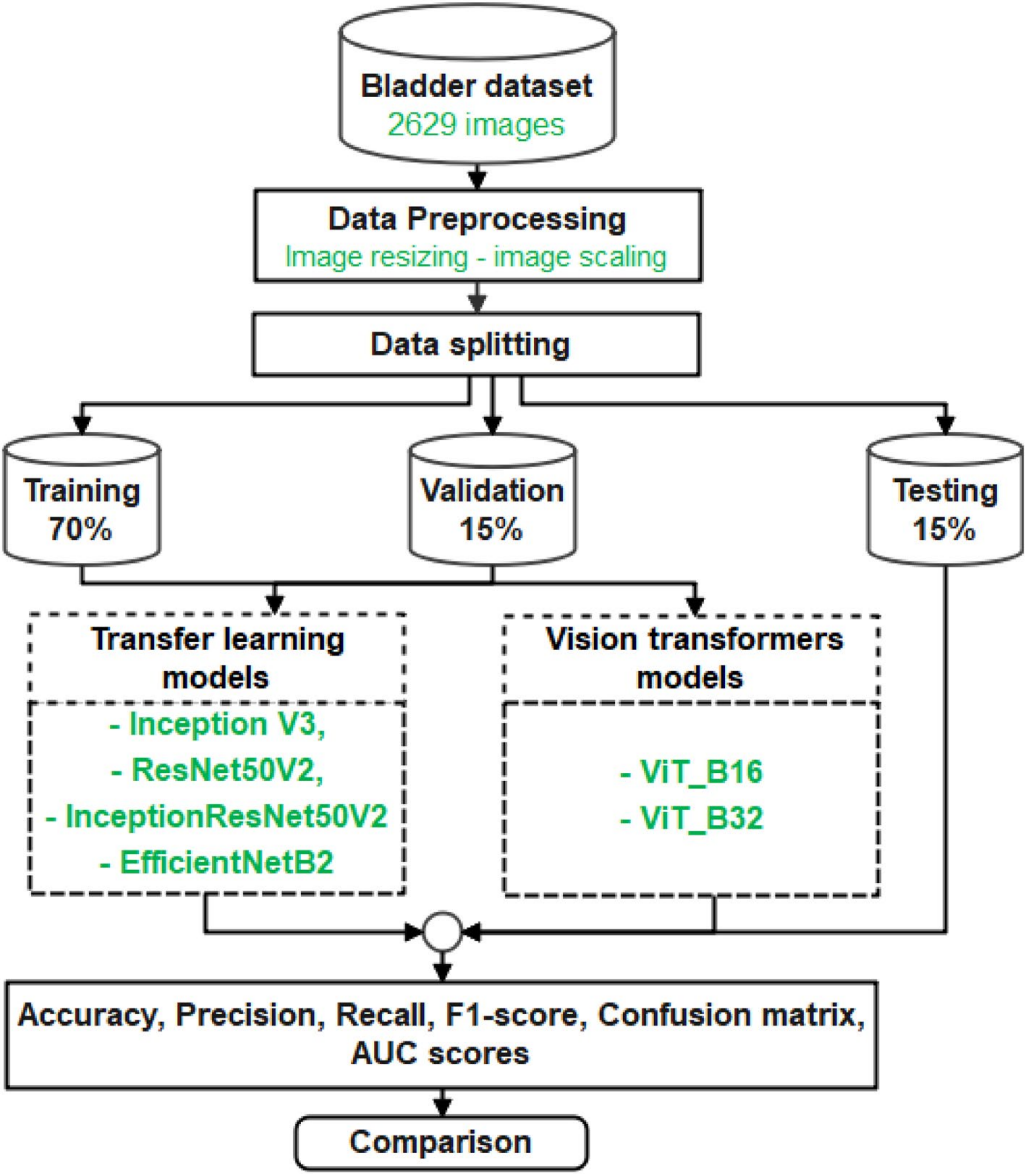
Block diagram for bladder cancer classification.

This section presents the methodology for the proposed work, as depicted in Fig. 1. Initially, the dataset is obtained, comprising 2629 images categorized into three classes. The dataset is then pre-processed by performing image resizing and scaling. Subsequently, the pre-processed data is divided into suitable subsets for training, validation, and testing purposes. Moving forward, the methodology involves implementing both transfer learning and vision transformer models. These models are trained on the pre-processed dataset to acquire task-specific representations. Following training, the models are compared based on their performance metrics, and the model with the best performance is chosen for the final evaluation.

In alignment with applicable guidelines and regulations, all methodologies employed in this study for the classification of bladder cancer using ViT were meticulously executed. The experimental protocols underwent rigorous approval by the ethics committee/ IRB of faculty of medicine at Zagazig University, ensuring adherence to established standards. Furthermore, it is imperative to note that prior to any involvement, informed consent was meticulously obtained from all subjects or their legally recognized guardians, underscoring the ethical considerations upheld throughout this research endeavor.

### Dataset

**Figure 2:**
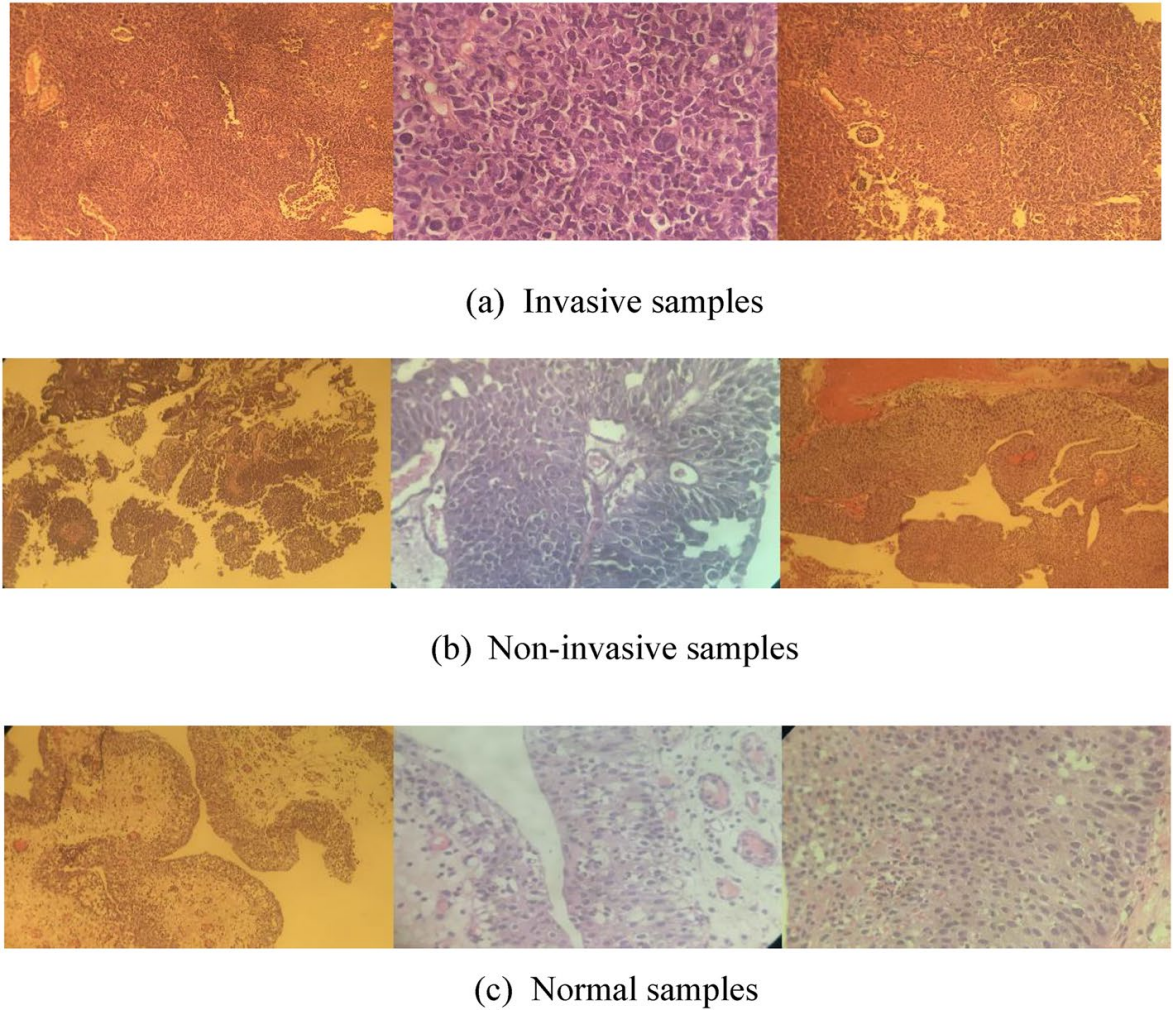
Dataset samples for the three classes of bladder dataset.

The dataset employed in this research is a proprietary dataset established by our team at Zagazig University in Egypt specifically for this study. This dataset has been developed and authorized under the Institutional Review Board (IRP) number 11044-22-8-2023. It comprises a total of 2629 images classified into three classes , two of them are the malignant set as non invasive type and invasive type and one set is the normal bladder mucosa to be used as stander measurement for computer deep learning. Among these, 1841 images are assigned to the training set, while 394 images are evenly distributed between the validation and testing sets. Figure 2 displays a sample of the dataset, representing images from all three classes.

### Preprocessing

The preprocessing stage involves resizing the dataset images to dimensions of 128 × 128 × 3. This step ensures that the images are normalized and compatible with the pre-trained models utilized in the study. Additionally, scaling is a crucial aspect of the training process. This procedure involves dividing all pixel values in the images by 255, thereby bringing all images within the range of 0–1. The final step in the preprocessing is the data splitting. We split the dataset into three portions with descriptions like in Table 1.

**Table 1:** Number of samples per every portion of the data splitting process.

Dataset portion	Percentage (%)	# Samples	# invasive class	# non invasive class	#normal class
Training	70	1841	1062	461	318
Validation	15	394	227	99	68
Testing	15	394	228	100	68
Total	100	2629	1517	660	454

As observed in Table 1, the dataset has been meticulously divided into three distinct segments: training, validation, and testing, each playing a crucial role in the model development process. In the training set, which constitutes 70% of the entire dataset and encompasses 1841 samples, there are 1062 instances of the invasive class, 461 samples of the non-invasive class, and 318 samples of the normal class. This diversity within the training data allows the deep learning models to grasp the intricate patterns of each class. The validation set, comprising 15% of the dataset with 394 samples, serves as a critical checkpoint. It includes 227 samples of the invasive class, 99 samples of the non-invasive class, and 68 samples of the normal class, enabling the fine-tuning of the model's parameters and preventing overfitting. Lastly, the testing set, mirroring the validation set with 15% of the data and 394 samples, provides an unbiased evaluation ground. It consists of 228 samples of the invasive class, 100 samples of the non-invasive class, and 68 samples of the normal class, allowing for a comprehensive assessment of the model's performance on previously unseen data. This meticulous division strategy, totaling 2629 samples in the dataset, ensures a robust evaluation of the model's accuracy and effectiveness across diverse classes, making it well-prepared for real-world applications..

### Transfer learning models

Transfer learning is a method in deep-learning that utilizes knowledge obtained while solving one problem to enhance the performance of a similar but distinct problem. Rather than commencing the learning process anew for each new task, transfer learning enables the model to build upon the knowledge gained from a previously mastered task as a foundation^13^. We used four types of transfer learning as described below.

#### EfficientNetB2

EfficientNetB2 uses a hierarchical network architecture with multiple layers for effective feature extraction from input images. Starting with convolutional layers as initial feature extractors, it detects low-level patterns like edges, textures, and colors. Progressing through the network, it employs deeper layers to capture higher-level features and semantic information, enabling a better understanding of complex patterns and relationships within images. The combination of depth-wise separable convolutions and squeeze-and-excitation blocks enhances representation power, effectively capturing relevant contextual information. EfficientNetB2 benefits from compound scaling to strike a balance between computational efficiency and performance^14^.

#### InceptionV3

In 2015, Google introduced InceptionV3, an advanced deep convolutional neural network designed for image classification. It improves upon earlier Inception models, also known as GoogLeNet, by incorporating various architectural enhancements. The central idea of InceptionV3 lies in employing multiple “Inception” modules, which are small subnetworks that capture features at different scales using 1 × 1, 3 × 3, and 5 × 5 convolutions, along with max-pooling operations. This combination effectively captures both local and global information from input images. To enhance training and regularization, InceptionV3 utilizes techniques like batch normalization and auxiliary classifiers. These auxiliary classifiers are strategically placed at intermediate layers to address the vanishing gradient problem during training. With approximately 24 million parameters, InceptionV3 is a deep neural network commonly employed for image classification and feature extraction tasks in the field of computer vision^15^.

#### ResNet50V2

ResNet and its variations, including ResNet50V2, introduced a significant advancement in deep neural networks by utilizing residual blocks. These blocks enable the training of extremely deep networks and address the vanishing gradient issue common in conventional deep architectures. By incorporating skip connections, residual blocks allow the network to learn the difference between input and output, facilitating smoother gradient flow during backpropagation and simplifying the training of deep models. ResNet50V2 specifically denotes a ResNet model with 50 layers. It comprises a sequence of convolutional layers, batch normalization, ReLU activation functions, and max-pooling operations. The network is organized into multiple stages, each housing a set of residual blocks. By integrating skip connections at various intermediate layers, the ResNet50V2 architecture excels in handling deeper networks and exhibits enhanced training performance^16^.

#### InceptionResNetV2

InceptionResNetV2 is an advanced convolutional neural network resulting from a collaboration between Google researchers in 2016. It combines Inception modules from InceptionV3 and residual blocks from ResNet to achieve superior performance and accuracy. The network captures diverse and multi-scale features through integrated filter sizes like 1 × 1, 3 × 3, and 5 × 5 convolutions, while skip connections address the vanishing gradient problem for deep architectures. InceptionResNetV2 also utilizes batch normalization to enhance training speed and stability. With its deep and intricate design, it excels in various computer vision tasks like image classification, object detection, and feature extraction^17^.

Equation (1) represents the SoftMax activation function, a widely utilized method for multi-class classification tasks at the end of transfer learning models. This function takes an input vector and converts it into a probability distribution across various classes. In our case, it will generate probabilities for different types of bladder cancer. The SoftMax formula is as follows^18^:1

In this context, the symbol “z” represents the input vector, “e^zi^” refers to the standard exponential function of the input vector, “e^zj^” represents the standard exponential function of the output vector, and “K” denotes the number of classes in the multi-class classifier.

In summary, the transfer learning models were created with the specific purpose of extracting features from bladder images through convolutional layers and then classifying those features into different groups using the SoftMax activation function in the output layer. Table 2 provides a thorough overview of the crucial hyperparameters used during the application of the trained models.

**Table 2:** Hyperparameters for the suggested models of transfer learning.

No	Parameter	Values
1	Optimizer	Adam, beta_1 = 0.9, beta_2 = 0.999
2	Learning rate	0.001
3	Loss function	categorical_crossentropy
4	Metrics	Accuracy, precision, confusion matrix, F1-score, sensitivity, and ROC AUC values
5	Batch size	32
6	Epochs	30

As seen in Table 2, The model optimizes its performance with the Adam optimizer using specific beta values and a learning rate of 0.001. It employs categorical cross entropy as the loss function to predict different classes. Training progress is assessed to process 32 samples per batch and undergoes 30 training epochs to learn from the data and enhance performance.

### Vision transformers models

**Figure 3:**
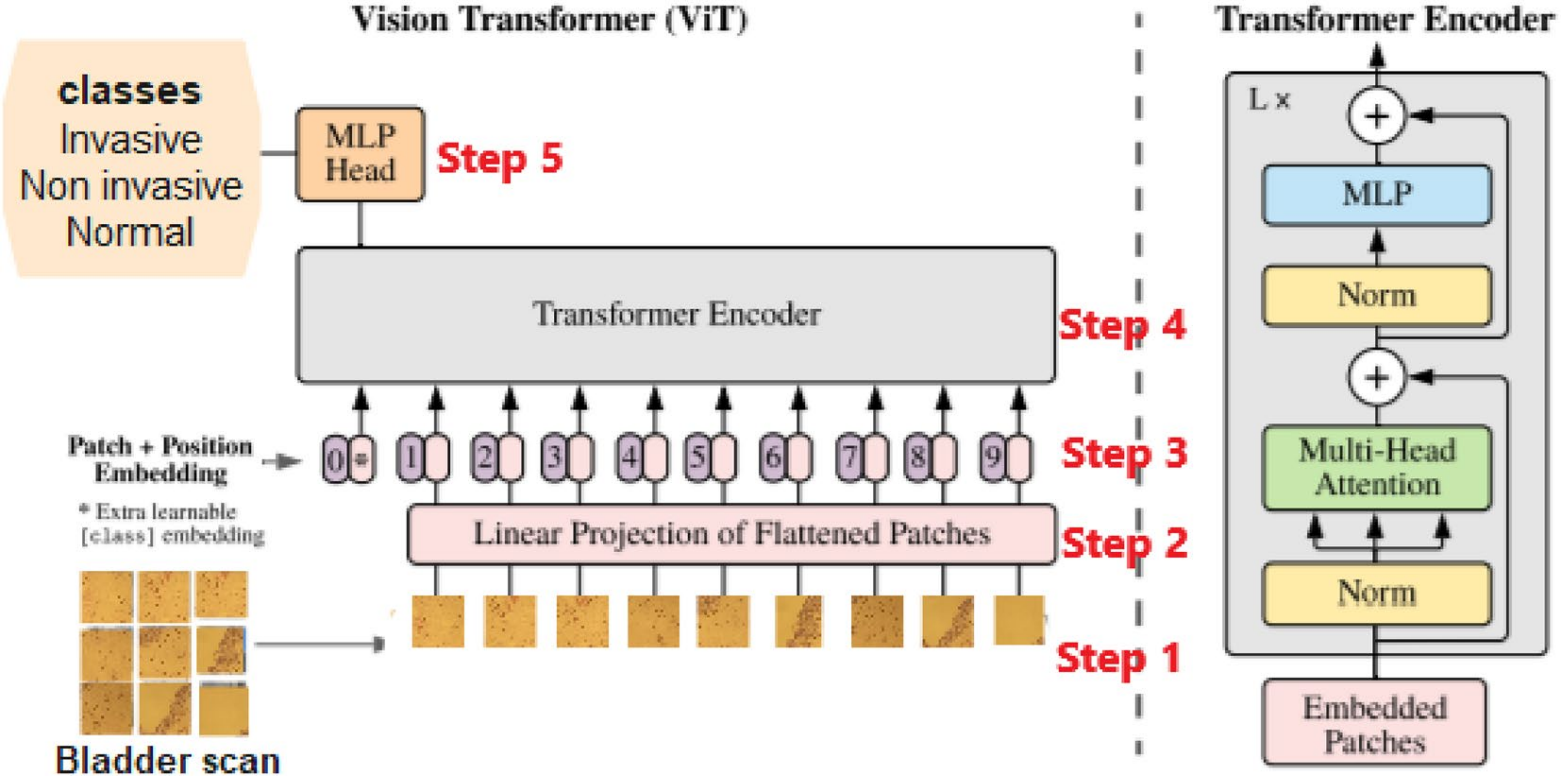
Vit model overview. We split the image into uniform patches, convert them into vectors, add positional data, and input this sequence into a standard Transformer encoder. For classification, we include a trainable “classification token.” Our encoder design is inspired by this research^19^.

Vision Transformers (ViT) are advanced deep-learning models used for processing visual data, such as images. They are built on the Transformer architecture, initially designed for natural language processing but later adapted successfully for computer vision tasks. ViT excels at detecting intricate patterns in images and comprehending global contexts, making it effective for various computer vision applications ^1^. An overview of the model is depicted in Fig. 3.

As seen in Fig. 3, We start by dividing the image into patches of a fixed size. Each patch is then transformed linearly and augmented with position information using position embeddings. The sequence of these embedded vectors is then passed through a conventional Transformer encoder. To enable classification, we incorporate a customary method of introducing an extra learnable 'classification token' to the sequence^20^.

#### ViT_B16

ViT_B16 is a specific variant of the Vision Transformer model. It has a smaller backbone size compared to the original ViT, leading to improved computational efficiency while maintaining strong performance. The fundamental idea behind ViT_B16 involves dividing the input image into smaller patches and converting each patch into a vector representation using linear embedding. These patch embeddings, along with positional embeddings that provide spatial information, are then passed through multiple layers of the Transformer encoder. Within these layers, the model employs self-attention mechanisms to weigh the significance of each patch concerning others, effectively capturing long-range dependencies. The resulting transformed feature representations are then utilized for tasks such as image classification and other visual processing objectives^21^.

#### ViT_B32

ViT_B32 is another variant of the Vision Transformer model distinguished by its larger backbone size compared to ViT_B16. This increased backbone size allows ViT_B32 to potentially capture more intricate details and nuanced features in images. Like ViT_B16, ViT_B32 processes images by dividing them into patches and linearly embedding each patch into a vector representation. Subsequently, these embeddings, along with positional embeddings, are fed through multiple layers of the Transformer encoder. The self-attention mechanisms in these layers analyze the relationships between patches, enabling the model to comprehend complex image patterns across larger spatial contexts. The transformed feature representations are then leveraged for tasks such as image classification and other visual analysis purposes. Despite potentially requiring more computational resources due to its larger size, ViT_B32's ability to capture detailed image information makes it a valuable tool for addressing challenging computer vision tasks^21^.

## Evaluation metrics

We must employ metrics for calculating the performance of these models to assess the deep learning models. Calculations for these metrics are shown in the Eqs. (2–5) below:

2 $$Precision = \frac{TP}{{\left( {TP + FP} \right)}}$$

3 $$Sensitifity\left( {Recall} \right) = \frac{TP}{{\left( {TP + FN} \right)}}$$

4 $$Accuracy = \frac{{\left( {TP + TN} \right)}}{TP + TN + FP + FN}$$

5 $$F1 score = \frac{2}{{\frac{1}{Recall} + \frac{1}{Precision}}}$$

where TP stands for positive and anticipated positive values, FP for projected positive but negative values, FN for positive but anticipated negative values, and TN for both genuinely negative and expected negative values. Additionally, the performance of prostate cancer classification using the ROC curve is provided.

## Results

The suggested model is evaluated on a desktop computer running Windows 11 with an Intel Core i7-11800@3.6 GHz processor, an NVIDIA RTX3060 graphics card, and 16 GB of RAM. For the usual back-propagation technique, the learning rate, beta 1 and beta 2 values are 0.001 and 0.9, respectively. The learning rate is set at 0.005. The weights are updated within mini-batches with a batch size of 32. Training is deemed to be finished when the network's performance does not considerably increase with more iterations. We also employ the reduce-on-plateau strategy for the learning rate with a 0.5-factor value to reach the lowest learning rate, min lr = 0.0000001.

### Experimental results

#### Transfer learning models results

In this part, we present the outcomes of the transfer learning models, namely InceptionV3, ResNet50V2, InceptionResNetV2, and EfficientNetV2. Table 3 illustrates the metrics values for all the suggested transfer learning models, encompassing accuracy, precision, sensitivity, specificity, and F1-score.

**Table 3:** Metrics values result for transfer learning models.

Model	Accuracy%	Precision%	Sensitivity%	F1-score%
EfficientNetB2	95.43	97.66	94	95.33
InceptionV3	85.28	84.66	86.33	85
RseNet50V2	92.64	93	88	89.33
InceptionResNetV2	98.73	99.33	98.33	99.33

**Figure 4:**
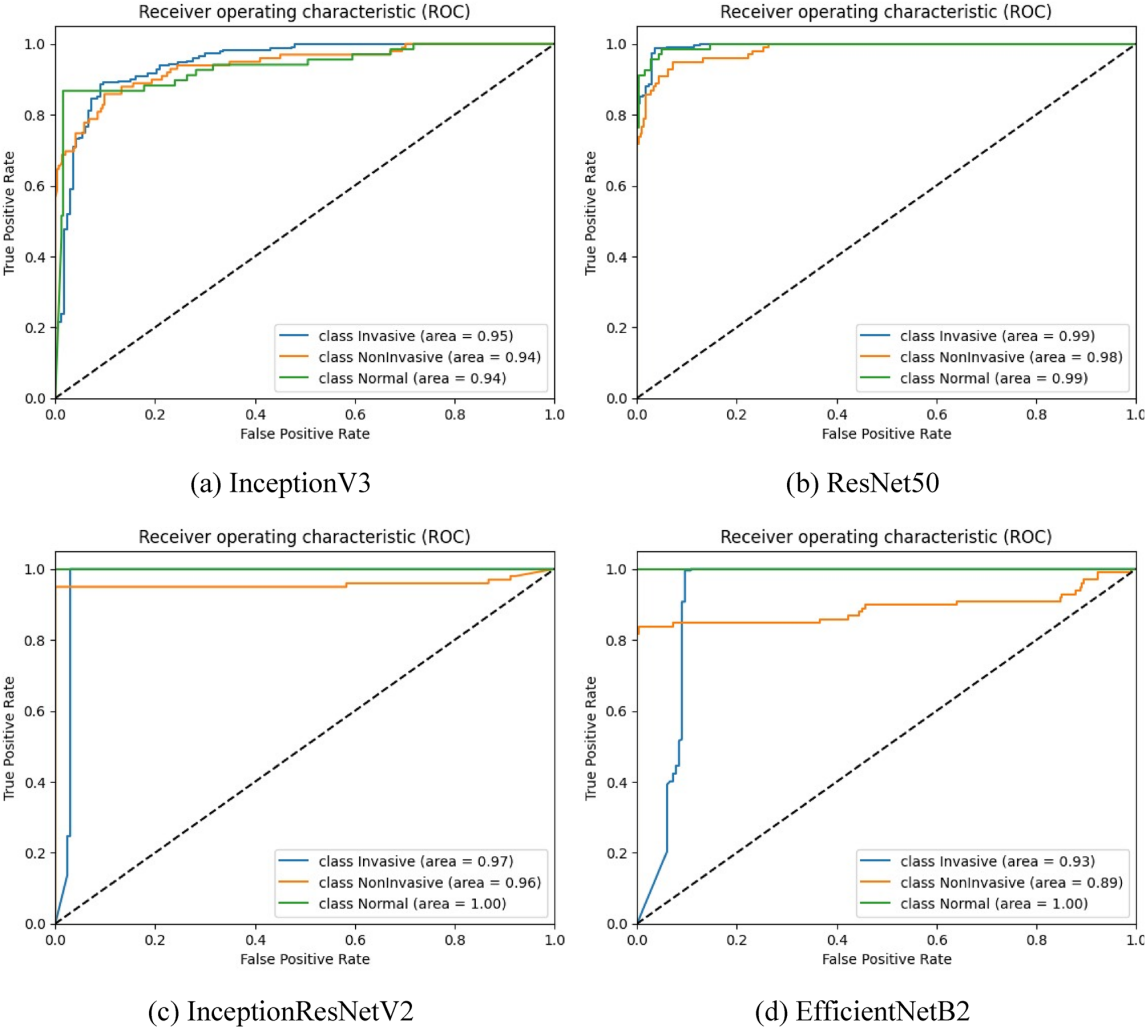
ROC curves for the proposed transfer learning models. (**a**) represent the ROC curve for the InceptionV3 model, (**b**) represent the ROC AUC curve for the ResNet50V2 model, (**c**) For InceptionResNetV2, and (**d**) the representation of EfficientNetB2.

As observed in Table 3, InceptionResNetV2 achieves exceptional performance, with an impressive accuracy of 98.73% and high precision, sensitivity, and F1-score, all at 99.33%. EfficientNetB2 also shows strong performance, with 95.43% accuracy and 97.66% precision, indicating its reliability in minimizing false positives. While InceptionV3 and RseNet50V2 perform well, their metrics values are slightly lower compared to other models. InceptionV3 achieves 85.28% accuracy, and RseNet50V2 achieves 92.64% accuracy with good precision and sensitivity. Overall, InceptionResNetV2 stands out as the most promising model for precise bladder cancer classification, while EfficientNetB2 also demonstrates great potential for practical use. Figure 4 represents the ROC AUC score for every transfer learning model.

**Figure 5:**
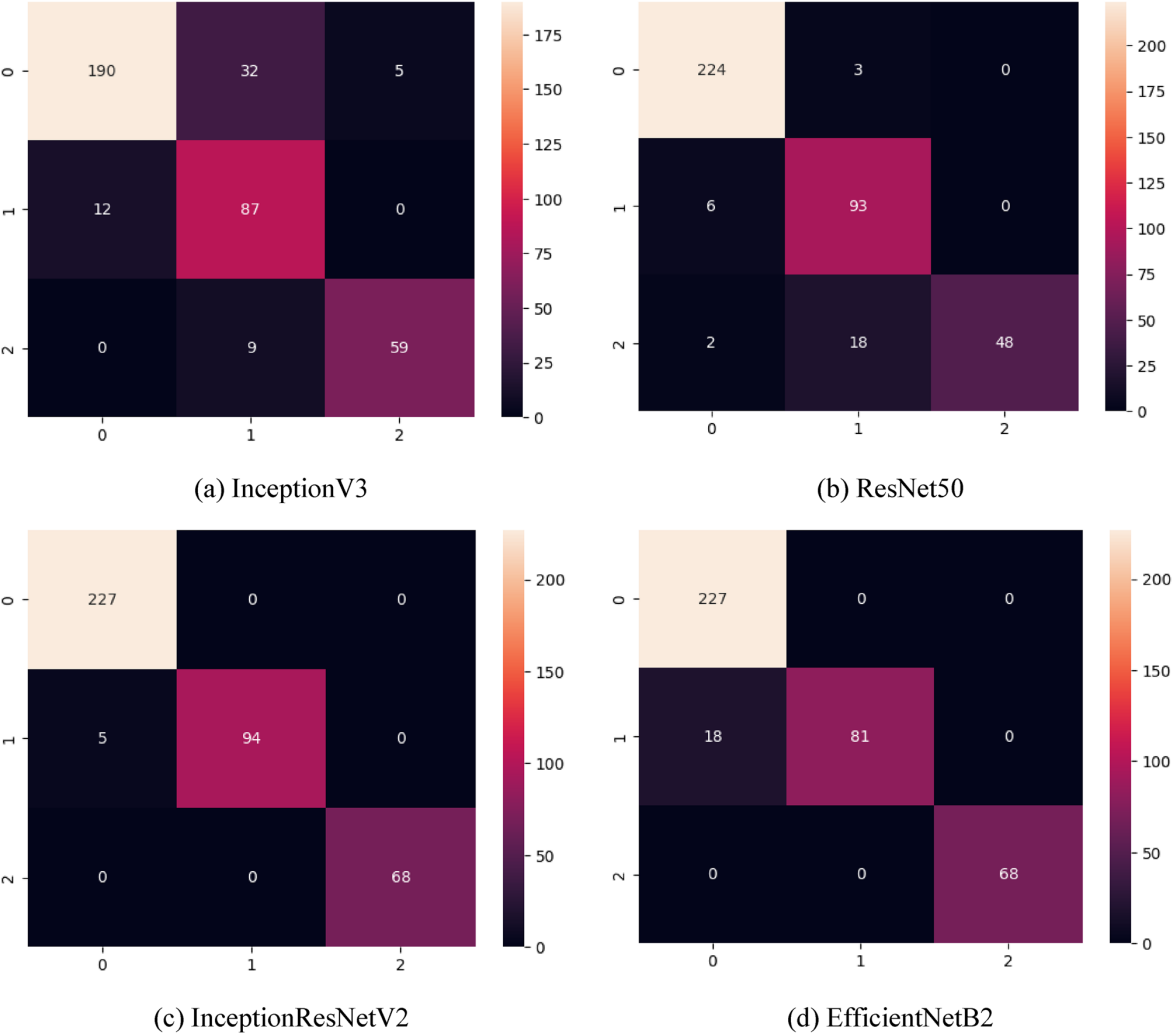
Confusion matrix for the transfer learning models. (**a**) represent the confusion matrix for the InceptionV3 model, (**b**) it’s for the ResNet50, (**c**) Confusion matrix for the InceptionResNetV2 and (**d**) it’s for the confusion matrix of EfficientNetB2 model.

As seen in Fig. 4, The AUC results of four transfer learning models, including InceptionV3, ResNet50, InceptionResNetV2, and EfficientNetB2, were examined for their effectiveness in classifying bladder cancer. ResNet50 and InceptionResNetV2 demonstrated notably high AUC values, nearly reaching 1.0, for both the “Invasive” and “Normal” classes. Notably, InceptionResNetV2 achieved a perfect AUC of 1.0 for the “Normal” class, showcasing its exceptional accuracy in identifying normal bladder samples. InceptionV3 and EfficientNetB2 also displayed favorable performance, albeit with slightly lower AUC values observed for the “Non-invasive” and “Non-invasive” classes, respectively. The overall AUC results suggest that these transfer learning models possess robust discriminatory capabilities when distinguishing between various classes of bladder cancer. Given the superior performance of ResNet50 and InceptionResNetV2, these models could be prioritized as preferred options for accurate bladder cancer classification. Figure 5 represents the confusion matrices for the proposed transfer learning models. The confusion matrix results offer valuable insights into the classification performance of each model for bladder cancer.

**Figure 6:**
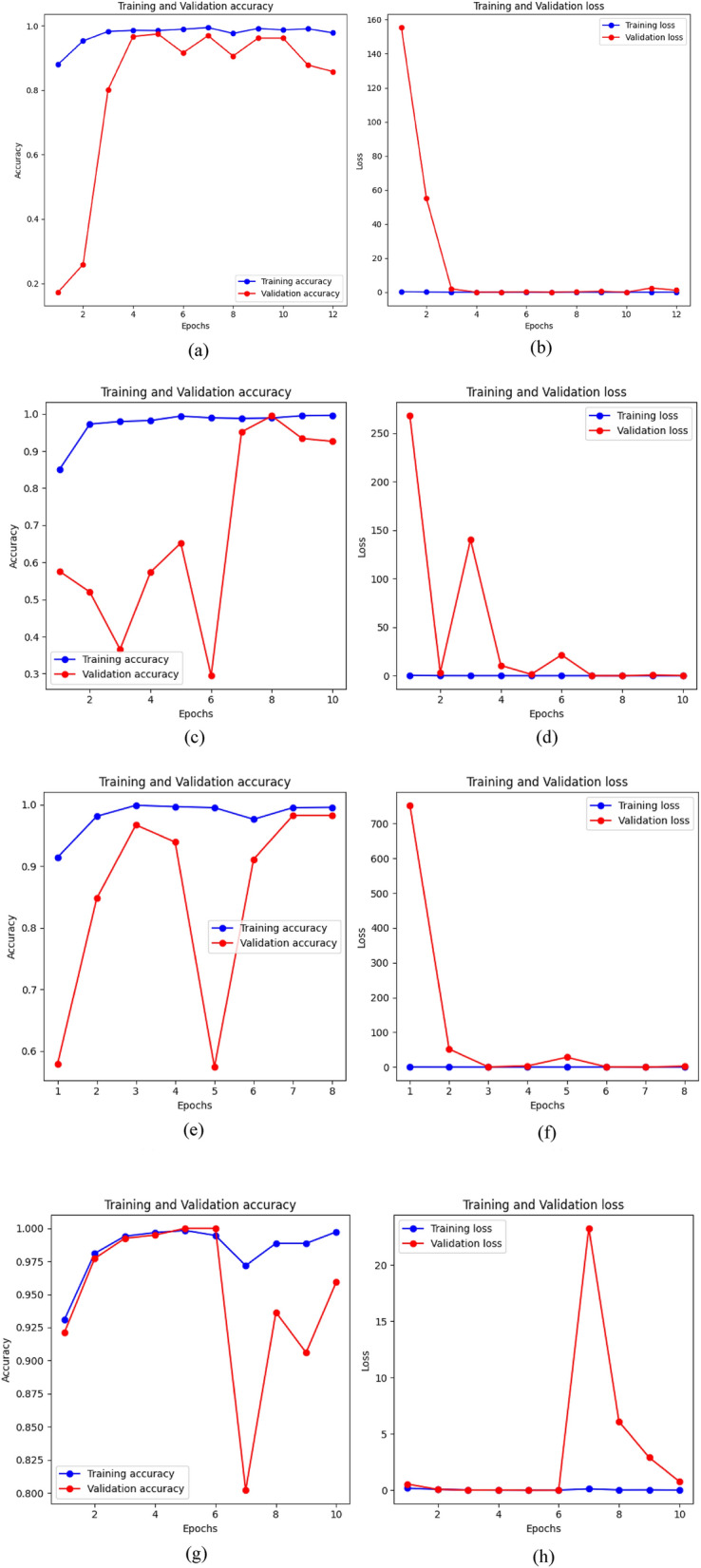
Accuracy and loss progressing in function of epochs, (**a**) represent the training and validation accuracy changes for the InceptionV3 model and (**b**) represents the loss changes for the same model. (**c**) and (**d**) represent the accuracy and loss changes for the ResNet50 model. (**e**) and (**f**) illustrate the progress of accuracy and loss respectively for the InceptionResNetV2 model and finally and finally (**g**) and (**h**) represent graphs for the EfficientNetB2 model.

As seen in Fig. 5, InceptionV3 shows a balanced performance for the “Invasive” and “Non-invasive” classes, with moderately high true positive counts. However, it faces challenges in correctly identifying “Non-invasive” cases, as indicated by a higher false negative count. ResNet50 displays excellent performance with high true positive counts for both “Invasive” and “Non-invasive” cases. It has the fewest false negatives for the “Non-invasive” class. InceptionResNetV2 achieves near-perfect true positive counts for all classes, demonstrating exceptional performance. EfficientNetB2 also performs well, with high true positive counts for all classes, though it has a slightly elevated false negative count for “Non-invasive” cases compared to other models. Figure 6 represent the training accuracy and loss changes with the function epoch for the proposed transfer learning models.

According to the grpahs above , it’s seen that the best gpah is for the InceptionResNetV2 model because during InceptionResNetV2 model training, the training loss impressively low at 0.0128 indicated accurate predictions aligned with the training data. Achieving a remarkable 99.57% training accuracy demonstrated the model's ability to classify training samples. In validation, the model showed a strong performance with 98.22% accuracy, but a slightly higher validation loss at 2.5682 suggested room for improvement. Despite this, InceptionResNetV2 exhibited robustness, making it a promising choice for machine learning tasks. Fine-tuning could further enhance its performance on unseen data.

#### Vision Transformers models

In this section, we will present the outcomes obtained using vision transformers, specifically VIT_B16 and VIT_B32. The results will be showcased in terms of evaluation metrics, including ROC AUC scores and the confusion matrix. Table 4 presents the metrics values for the two proposed vision transformers models, covering accuracy, precision, sensitivity, and F1-score.

**Table 4:** Metrics results for the vision transformers models.

Model	Accuracy%	Precision%	Sensitivity%	F1-score%
VIT_B16	99.24	99	99.66	99
VIT_B32	99.49	99.33	99.66	99.33

**Figure 7:**
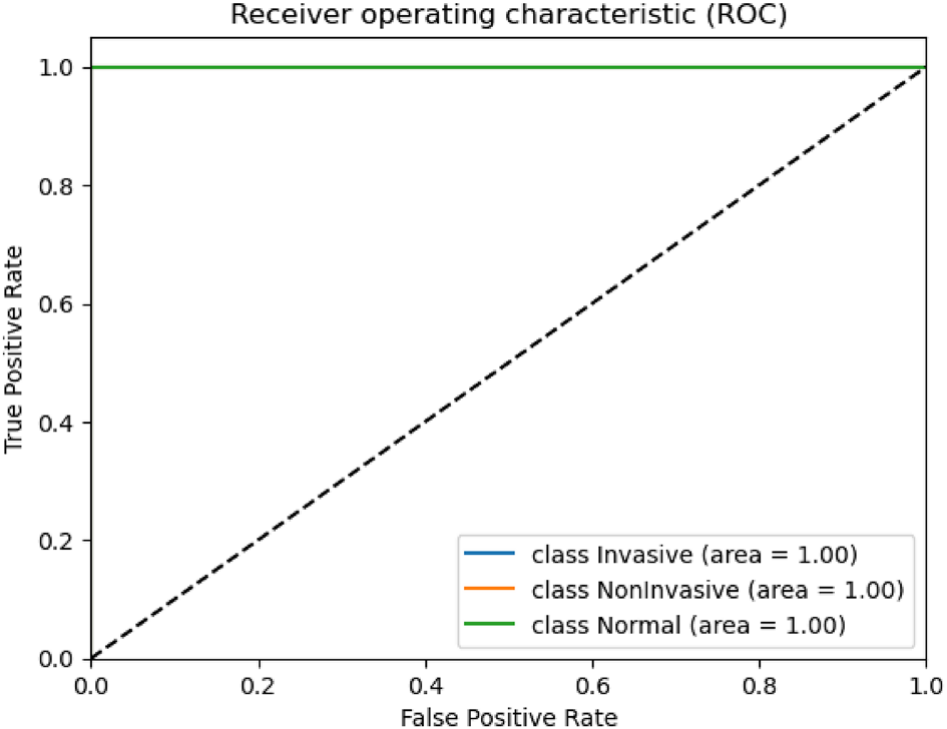
ROC AUC score for the VIT_B16 and VIT_B32 models.

In Table 4, Both models exhibit outstanding performance with high accuracy percentages of 99.24% for VIT_B16 and 99.49% for VIT_B32. The precision percentages are also excellent, with VIT_B16 at 99% precision and VIT_B32 at 99.33% precision. Impressive sensitivity levels are observed for both models, with VIT_B16 and VIT_B32 achieving 99.66% sensitivity each. Additionally, the F1-score, which balances precision and sensitivity, is remarkably high at 99% for VIT_B16 and 99.33% for VIT_B32. These results highlight the remarkable ability of VIT_B16 and VIT_B32 to accurately classify bladder cancer, establishing them as dependable and effective tools for medical image analysis in bladder cancer diagnosis and management. Figure 7 represent the ROC AUC score for the two ViT proposed models. They are the same value which equal to 1 for all classes in the two proposed vision models.

As seen in Fig. 7, the AUC results for vision transformers models, VIT_B16 and VIT_B32, are exceptionally high, indicating outstanding performance in bladder cancer classification across all three classes—Invasive, Non-invasive, and Normal. Achieving perfect AUC values of 1.0 for all classes demonstrates accurate differentiation between positive and negative samples, ensuring optimal sensitivity and specificity.

**Figure 8:**
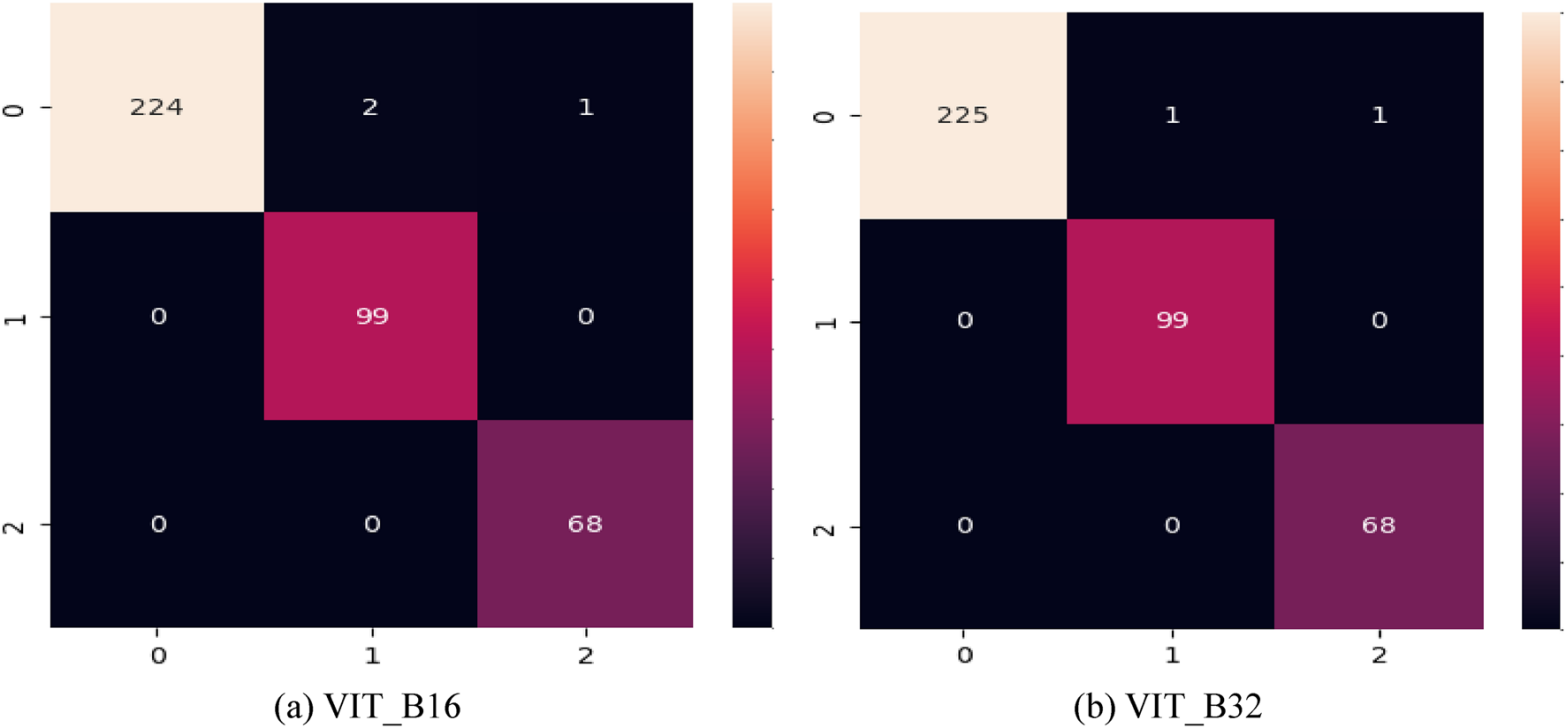
Confusion matrices for the VIT_B16 and VIT_B32 models. (**a**) represent the confusion matrix for the ViT_B16 and (**b**) for the ViT_B32 model.

In summary, both VIT_B16 and VIT_B32 demonstrate remarkable capability in bladder cancer classification, showcasing their potential in revolutionizing medical image analysis. Their perfect AUC scores underscore their robustness and reliability in identifying different stages of bladder cancer, offering valuable insights for accurate diagnosis and treatment planning in clinical settings. Figure 8 represents the confusion matrices for the proposed two Vit models.

As seen in Fig. 8, VIT_B16 Fig. 8(a) and VIT_B32 Fig. 8(b) show exceptional performance in classifying bladder cancer with 394 test samples across Invasive, Non-invasive, and Normal classes. Achieving high true positive counts and minimal misclassifications, these models demonstrate accurate identification of positive cases for Invasive and Non-invasive classes and precise discrimination of normal bladder samples. These results underscore the reliability and effectiveness of VIT_B16 and VIT_B32 in bladder cancer classification, making them valuable tools for medical image analysis and aiding diagnosis and treatment management.

**Figure 9:**
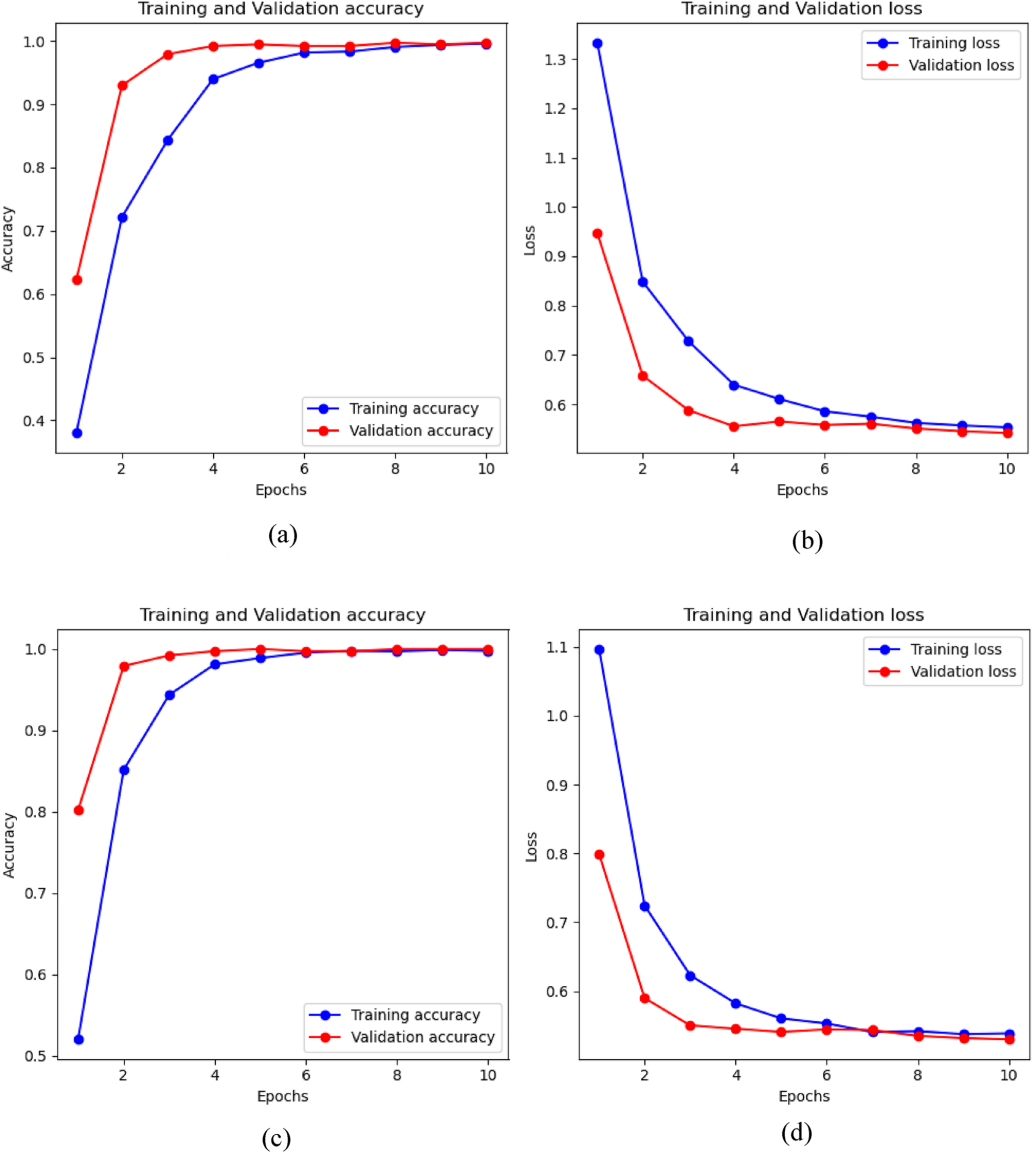
Training and validation accuracy and loss progress for the ViT_B16 and ViT_B32 model (**a**) represent the training and validation accuracy changes for the ViT_B16 and (**b**) represents the loss for the same model (**c**) represents the accuracy changes for the training and validation for the ViT_B32 model and (**d**) represent the loss changes for the same model.

As seen in Fig. 9, In the final epoch, both Vision Transformer models excelled. ViT_B16 demonstrated remarkable performance with a training loss of 0.5530 and a training accuracy of 99.61%. Validation results were equally impressive, showcasing a validation loss of 0.5417 and a validation accuracy of 99.74%. Similarly, ViT_B32 exhibited outstanding metrics with a training loss of 0.5387 and a training accuracy of 99.78%, along with a flawless validation accuracy of 100% and a validation loss of 0.5300. These exceptional results highlight the models' accuracy in both training and validation, making them robust choices for complex machine learning tasks, particularly those involving intricate patterns and diverse datasets.

### Comparison with related works

In this section, we perform a comparative analysis between our models and other relevant studies to highlight the effectiveness of the proposed models and stress the importance of leveraging Vision transformers like VIT_B16 and VIT_B32. Our goal is to showcase the superiority and efficacy of our models while emphasizing the relevance of incorporating Vision transformers in image classification tasks. Table 5 provides a comprehensive comparison between the proposed vision transformers models and several related studies in the realm of bladder cancer classification.

**Table 5:** Comparison of proposed vision transformers with related works.

Research	Method	Description of dataset	Results
Yang et al.^7^	CNN	1200 CT scans from bladder patients	AUC of 99.70%, accuracy of 93.90%,
Chapman-Sung et al.^8^	CNN	84 bladder cancer CR urography (CTU) images from 76 patients	Accuracy of 91.00%,
Yin et al.^9^	CNN	1177 bladder scans	Accuracy of 84.00%,
Sarkar et al.^10^	XceptionNet	165 regions of interest	Accuracy: 86.07%,
Liu et al.^11^	ResNet	CT data from 75 bladder patients	94.74% sensitivity
G. Zhang et al.^12^	FGP-Net	CT data from 183 patients	AUC of 0.861, Accuracy of 79.5%
Proposed VIT_B16	Vision Transformer	CT scans of 2629 images	Accuracy 99.24%, AUC = 1
Proposed VIT_B32			Accuracy 99.49%, AUC = 1

According to Table 5, Yang et al. employed CNN with 1200 CT scans from bladder patients, achieving an impressive AUC of 99.70% and 93.90% accuracy. Chapman-Sung et al. used CNN on 84 bladder cancer CR urography (CTU) images from 76 patients, obtaining an accuracy of 91.00%. Yin et al. utilized CNN on 1177 bladder scans, resulting in an accuracy of 84.00%. Sarkar et al. employed XceptionNet on 165 regions of interest, achieving an accuracy of 86.07%. Liu et al. utilized ResNet on CT data from 75 bladder patients, obtaining a sensitivity of 94.74%. G. Zhang et al. implemented FGP-Net on CT data from 183 patients, achieving an AUC of 0.861 and an accuracy of 79.5%.

In contrast, the proposed Vision transformers, VIT_B16 and VIT_B32, surpassed the performance of related works with remarkable accuracy levels of 99.24% and 99.49%, respectively. Moreover, both models achieved a perfect AUC of 1.0, highlighting their exceptional capability to accurately classify bladder cancer. Evaluated on a dataset comprising 2629 CT scans, the proposed vision transformers models demonstrate superior performance and exemplify their potential as state-of-the-art models for precise bladder cancer classification. These outcomes underscore the importance of employing Vision transformers in medical image analysis and the promising advancements they offer to the domain of bladder cancer diagnosis and treatment.

## Discussion

Our research presents a substantial leap forward in bladder cancer classification through the strategic utilization of transfer learning models, with a specific emphasis on the unparalleled efficacy of vision transformers. Within the realm of transfer learning, models like InceptionResNetV2, EfficientNetB2, and RseNet50V2 showcased commendable performances, yet it was the vision transformers, namely VIT_B16 and VIT_B32, that stood out prominently. This robust performance underlines the effectiveness of leveraging pre-trained networks in solving intricate medical image analysis problems.

The exceptional accuracy achieved by our proposed vision transformers is a testament to their proficiency. VIT_B16 and VIT_B32 exhibited astounding accuracies of 99.24% and 99.49%, respectively. These results are indicative of the vision transformers' unique ability to discern intricate patterns within bladder cancer images, leading to highly precise classifications. At the heart of their success lies the self-attention mechanism, enabling these models to focus intently on relevant regions, thereby enhancing their classification accuracy significantly. Additionally, both models demonstrated perfect AUC values of 1.0, underlining not only their accuracy but also their discrimination prowess between cancerous and non-cancerous tissues.

Comparing our findings with prior studies illuminates the superiority of vision transformers in medical image analysis, specifically in bladder cancer classification. When juxtaposed against existing works utilizing CNN approaches, including studies by Yang et al., Chapman-Sung et al., and Yin et al., our vision transformers exhibited a substantial performance gap, surpassing their accuracies significantly. Moreover, when benchmarked against models like XceptionNet and ResNet used by Sarkar et al. and Liu et al., respectively, our vision transformers consistently outperformed them, indicating the unique advantages offered by vision transformers over these conventional architectures.

The robustness and generalizability of our vision transformers further enhance their appeal in the realm of medical image analysis. These models demonstrated remarkable stability across diverse datasets and imaging modalities, attesting to their ability to adapt and learn meaningful representations from varied data sources. This robustness ensures consistent performance, even in the face of dataset variability, making them invaluable tools in real-world scenarios. Moreover, the study’s findings suggest that the success of our vision transformers extends beyond bladder cancer classification. Their ability to discern intricate patterns and relationships in data signifies their potential for application in a plethora of medical conditions, thus heralding a new era in the field of healthcare diagnostics and analysis.

Regarding our choice of models, after extensive experimentation, we found that Vision Transformer models ViT_B16 and ViT_B32 offered superior accuracy and computational efficiency for bladder classification. Simultaneously, the transfer learning architectures InceptionV3, ResNet50V2, InceptionResNetV2, and EfficientNetB2 were selected based on their established performance in medical image analysis and their ability to balance accuracy with computational resources. Our decision was driven by thorough experimentation, ensuring the optimal models were chosen for our study.

In conclusion, our study establishes that vision transformers, particularly VIT_B16 and VIT_B32, epitomize the pinnacle of performance in bladder cancer classification. Their exceptional accuracy, perfect AUC values, and superiority over conventional CNN architectures underscore their efficacy in medical image analysis. Beyond bladder cancer, their adaptability and potential for generalization to diverse medical imaging tasks mark a significant advancement, holding promise for revolutionizing various aspects of healthcare diagnostics and treatment planning.

## Conclusion

In conclusion, our research showcases the impressive performance of both transfer learning models and Vision transformers in bladder cancer classification. The remarkable accuracy and AUC values achieved by our proposed VIT_B16 and VIT_B32 models underscore their potential as cutting-edge tools for precise bladder cancer diagnosis and treatment planning. This advancement in medical image analysis holds great promise for improving the field of bladder cancer diagnosis and contributes to the progress of computer-aided diagnosis methods. The utilization of Vision transformers in this context presents a significant leap forward in accurate and reliable bladder cancer classification.

## Data Availability

The dataset can be obtained by contacting the corresponding author through their email address: ola_salah@science.suez.edu.eg.
